# Draft Genome Sequence of *Pseudomonas moraviensis* R28-S

**DOI:** 10.1128/genomeA.00035-14

**Published:** 2014-02-20

**Authors:** Samuel S. Hunter, Hirokazu Yano, Wesley Loftie-Eaton, Julie Hughes, Leen De Gelder, Pieter Stragier, Paul De Vos, Matthew L. Settles, Eva M. Top

**Affiliations:** a Department of Biological Sciences, University of Idaho, Moscow, Idaho, USA; b Institute for Bioinformatics and Evolutionary Studies (IBEST), University of Idaho, Moscow, Idaho, USA; c Laboratory of Microbiology, Ghent University, Ghent, Belgium

## Abstract

We report the draft genome sequence of *Pseudomonas moraviensis* R28-S, isolated from the municipal wastewater treatment plant of Moscow, ID. The strain carries a native mercury resistance plasmid, poorly maintains introduced IncP-1 antibiotic resistance plasmids, and has been useful for studying the evolution of plasmid host range and stability.

## GENOME ANNOUNCEMENT

*Pseudomonas moraviensis* R28 is a member of the *Gammaproteobacteria* and was originally reported as *Pseudomonas koreensis* R28. The strain was isolated from activated sludge of the municipal wastewater treatment plant in Moscow, ID, as a transconjugant after a plate mating of a sludge sample with a donor plasmid pB10::*rfp*. The transconjugants were selected on defined aerobic basal (DAB) medium supplemented with succinate, acetate, and citrate and the antibiotics tetracycline (10 mg/liter) and streptomycin (50 mg/liter) (1). In the laboratory, it has been a useful strain for studying the stability and evolution of broad-host-range multidrug resistance plasmids (1–3). The first isolate of the species *P. moraviensis* was collected from oil-polluted soil in the Czech Republic and was shown to hydrolyze diverse carbohydrates and utilize an impressive array of substrates (4). Strain R28-S, a streptomycin-resistant mutant of R28, was identified as a member of this species via an in-house four-gene-based (*atpA*, *glnA*, *rpoB*, and *rpoD*) multilocus sequence analysis (MLSA) scheme, which for each gene undoubtedly showed the highest match with the type strain *P. moraviensis* LMG 24280.

The genome of *P. moraviensis* R28-S was sequenced using a whole-genome shotgun approach, with paired 150-bp reads generated on the MiSeq (Illumina) and 454 (Roche) sequencing platforms. The sequencing adapters and low-quality bases were trimmed using a custom script, and the reads were assembled using Newbler version 2.6. A total of 36 contigs >500 bp were produced. Of these, the largest is 815,593 bp and the N_50_ contig size is 462,409 bp. The assembled contigs were ordered and oriented using a whole-genome map produced by OpGen optical mapping MapIt services. The optical mapping results were corroborated by aligning R28-S contigs against the closely related and so-called *Pseudomonas fluorescens* Pf0-1 (accession no. NC_007492) genome using r2cat (5). Small contigs that could not be scaffolded with the optical map were placed using these alignments. Additional gaps were then closed using the program GapFiller (6), and the paired Illumina reads resulted in a final assembly consisting of 12 contigs with a total length of 6,226,470 bp (including estimated gap sizes).

Included in the set of contigs was a native 81,846-bp plasmid, pR28. The replication initiator gene (*repA*) and origin of replication gene (*oriV*) of pR28 bear 89 and 84% nucleotide identities, respectively, to that of the IncP-9θ plasmid pSVS15 isolated from *Pseudomonas putida* (7). Although many IncP-9 plasmids are self-transferable, pR28 does not encode a full conjugative system. Its genome contains multiple transposons, one of which encodes resistance to mercury. Based on its read coverage, its copy number is estimated at 2/cell (1.9× for each chromosome copy).

### Nucleotide sequence accession numbers.

This whole-genome shotgun project has been deposited at GenBank under the accession no. AYMZ00000000. The version described in this paper is version AYMZ00000000.1. Strain R28-S is available from the LMG culture collection as LMG 28150 (http://bccm.belspo.be/about/lmg.php).
